# Using Behavior Change Interventions in Cardiac and Pulmonary Rehabilitation: Perspectives from Healthcare Professionals in the United Kingdom

**DOI:** 10.3390/ijerph19041980

**Published:** 2022-02-10

**Authors:** Eleanor M. Whittaker, Andrew R. Levy, Bashir Matata, Florence E. Kinnafick, Adrian W. Midgley

**Affiliations:** 1 Department of Psychology, Edge Hill University, Lancashire L39 4QP, UK; eleanor.whittaker@northyorks.gov.uk; 2 Health Research Institute, Edge Hill University, Lancashire L39 4QP, UK; midglead@edgehill.ac.uk; 3 Liverpool Heart and Chest Hospital NHS Foundation Trust, Liverpool L14 3PE, UK; matata_bashir@hotmail.com; 4 School of Sport, Exercise and Health Sciences, Loughborough University, Leicestershire LE11 3TU, UK; f.e.kinnafick@lboro.ac.uk; 5 Department of Sport and Physical Activity, Edge Hill University, Lancashire L39 4QP, UK

**Keywords:** qualitative, health, rehabilitation, behavior change

## Abstract

This study explores healthcare professionals’ experiences of using behavior change interventions in clinical practice. Semi-structured qualitative interviews were conducted with 11 healthcare professionals working in a cardiac and pulmonary rehabilitation National Health Service Trust in the United Kingdom. Interviews were transcribed and analyzed using inductive thematic analysis. Four overarching themes representing healthcare practitioners’ perceptions of using behavior change interventions were identified: (1) reliance on experiential learning, (2) knowledge transition, (3) existing professional development programs, and (4) barriers and facilitators for continued professional development. The results are discussed in relation to the implications they may have for behavior change training in clinical healthcare practice. Healthcare professionals require bespoke and formalized training to optimize their delivery of behavior change interventions in cardiac and pulmonary rehabilitation. Doing so will enhance intervention fidelity and implementation that can potentially ameliorate patient rehabilitation outcomes.

## 1. Introduction

The significant financial strain experienced by the National Health Service (NHS) in the United Kingdom and the increasing number of patients living with long-term health conditions has led to non-physician healthcare professionals expanding and diversifying their role to include public health advocacy [1]. Despite this role expansion being extremely common across a range of healthcare disciplines, healthcare professionals report feeling that their training in delivering behavior change as a component of their standard practice is poor [2]. This contributes to the perception that these additional responsibilities detract from their key roles [1]. There has been pessimism about the success of behavior change interventions and concern that attempting to discuss potentially sensitive topics, such as weight loss, may damage relationships with their patients [3]. Consequently, despite the potential of behavior change interventions to improve several public health concerns if properly embedded into routine clinical practice, healthcare professionals often make a cursory attempt at this or avoid it altogether [2]. Further research is needed to better understand healthcare professionals’ opinions and experiences to identify ways in which they can be assisted to incorporate topics such as behavior change into their standard practice. It is essential that research evidence can be translated into clinical practice to deliver safe, transparent, effective, and efficient healthcare provision [4]. However, there is currently a paucity of research exploring how behavior change interventions are utilized in health contexts, with limited progress made over the past decade [5].

In healthcare settings, behavior change interventions are a coordinated set of techniques used by health professionals to help patients change specified behaviors that promote better health. There has been a proliferation in research focusing on predicting, explaining, and promoting behavior change amongst a range of populations [6,7]. However, there is limited translation of this knowledge with the aim of affecting healthcare professionals’ practice [8], specifically for long-term conditions. Doctors, for example, are primarily trained to diagnose and treat medical conditions rather than monitoring and attempting to modify their patients’ behaviors and may be hesitant in delivering behavior change interventions within their standard practice [8]. This problem is further exacerbated by how only some and not all healthcare professionals receive behavior change training. Some healthcare professionals have expressed discomfort about discussing behavior change and frustration with patients’ failure to follow health advice [9]. The extent to which cardiac and pulmonary rehabilitation healthcare professionals understand and feel comfortable discussing behavior change, therefore, warrants further investigation. Additionally, despite theories of behavior change being increasingly applied to clinician behaviors such as prescribing practices, these models have not been tested rigorously in this setting. There is a lack of understanding of how behavior change theory should be applied in these clinical settings [10]. Indeed, it is not fully understood how cardiac and pulmonary rehabilitation professionals can play a more effective role in tackling patients’ health behaviors.

Within cardiac and pulmonary rehabilitation, behavior change aims to prevent a patient’s readmission to the service by developing a patient’s ability to self-manage their condition [11]. Self-management of chronic illness has been widely recognized to support patients to live the best possible quality of life with their chronic condition and is integral in facilitating secondary prevention [12]. Despite the effectiveness of self-management in facilitating secondary prevention, it is often difficult for patients to develop this ability. Therefore, cardiac and pulmonary rehabilitation occupies a unique space, where healthcare professionals can provide support at a teachable moment shortly after a stressful life event such as acute illness or hospitalization. It is at this vital moment that healthcare professionals can offer advice to patients about improving their health by changing their health-related behaviors [13]. Nurse-led interventions initiated during this time show promising results for patients with a range of chronic diseases [14], meaning, to a greater extent, there is scope for behavior change to be embedded within the cardiac and pulmonary rehabilitation domain.

Using a qualitative approach, the aim of this study is to investigate cardiac and pulmonary healthcare professionals’ real-world experiences of utilizing behavior change interventions as part of their clinical practice. The findings of this study will help inform the use of behavior change interventions that are acceptable for healthcare professionals working in cardiac and pulmonary rehabilitation.

## 2. Materials and Methods

### 2.1. Participants

Through purposeful sampling, 11 healthcare professionals (2 male and 9 female) working as part of multidisciplinary teams for a cardiac and pulmonary rehabilitation service in the United Kingdom participated in the study. The participants included cardiac nurses (*n* = 3), a matron (*n* = 1), exercise physiologists (*n* = 3), a healthcare assistant (*n* = 1), an assistant practitioner (*n* = 1), an exercise instructor (*n* = 1), and a physiotherapy assistant (*n* = 1). Healthcare professionals were informed of the intention to develop an intervention derived from their interview data and were invited to participate via email. All participants provided written consent, and the study was given favorable ethical opinion by the Northwest, Greater Manchester Research Ethics Committee (REC reference: 17/NW/0332; IRAS project ID: 226025).

### 2.2. Data Collection and Procedure

Data were collected by the first author using face-to-face semi-structured interviews in either a hospital seminar room or a private room in a leisure center, based on participant availability. To ensure discussion remained pertinent to the aims of the study, an interview guide was developed that allowed the interviewer to ensure the same coverage of topics across all participants. The interview guide was informed by a review of the literature and consultancy between the research team and senior healthcare professionals. A combination of open and closed questions was utilized. Open questions provided participants with the opportunity to discuss their lived experiences (e.g., “*What is your experiences of using behavior change techniques*”). Closed questions (e.g., “*Do you think it is part of your role to utilize behavior change knowledge*”), prompts, and probes were utilized to garner greater depth of responses (e.g., “*I am sensing that…*”; “*Could you elaborate…*”). Interviews were recorded using a Dictaphone that lasted between 23 and 81 min (M = 44 min), supplemented by addressing the aims of the study and assurances about confidentiality. In total, 13.5 h of interview data were collected.

### 2.3. Data Analysis

Data analysis followed Braun and Clarke’s thematic approach [15], including data familiarization, coding, and searching for and defining themes. This approach was adopted, as it provides a rich and detailed account of data [16]. Specifically, our analysis was inductive, meaning that the findings were strongly data-driven. A pre-existing coding frame was not adopted but, instead, themes were formed inductively throughout the concurrent processes of data collection and analysis. Through this concurrent process, themes and issues raised during earlier interviews informed the conduct of subsequent interviews. Miscellaneous themes that did not intuitively fit with the rest of the data were also recorded, described, and discussed by the research team. Both the first and second authors consulted, allowing comparison and reflection on theme identification to ensure our findings were credible. Further trustworthiness was established through the first author keeping a personalized reflexive journal throughout.

## 3. Results

### 3.1. Reliance on Experiential Learning

When questioned about their level of understanding of behavior change, healthcare professionals generally perceived motivational interviewing, goal setting, and behavior change to be synonymous, with several participants discussing motivational interviewing courses they had taken part in or “*SMART*” goal-setting techniques that they had utilized with patients (Healthcare Professional 6, line 586). Another professional discussed how changing behavior is “really, really hard” (line 930), but successfully changing behavior could be achieved by “*getting the trust, and you knowing your stuff. You’ve got to be 100 percent knowing your stuff because the patient can see right through that*” (Healthcare Professional 8, lines 930–932). This demonstrates that the intervention needs to equip healthcare professionals with the competence to deliver behavior change in a way that the patients will trust the messages they are providing. Healthcare professional 6 also highlighted barriers to changing patients’ behaviors by discussing the “*personality of the patient and their demographics and where they come from and their culture within the family*” as potential determinants of behavior change (Healthcare Professional 8, lines 926–927). This demonstrates that professionals are generally aware of behavior change but lack the skills to systematically implement these techniques within their practice. This lack of access to behavior change training is corroborated by healthcare professional 3:

*“I do think it’s (behavior change has) got its part. It’s definitely got its part hasn’t it? Yet again, you don’t get no training. It’s even when the patient—it’s only through experience you pick up little things”*

(Healthcare Professional 3, lines 229–231)

Given the lack of formalized training in behavior change that has been recognized in previous sections, healthcare professional 3 suggests that her competence in delivering behavior change is largely reliant on experiential learning. Across all healthcare professionals interviewed, healthcare professional 6 discussed his experience in behavior change and where this understanding stemmed from most extensively, suggesting that his university education provided him with his current levels of understanding of behavior change.

*“My understanding of behavior change is only what I did in university. I—it’s probably the—the self-efficacy model. I like to talk to people about their self-confidence and self-confidence in particular tasks”*

(Healthcare Professional 6, lines 165–181)

Due to their perceived limited understanding of behavior change in rehabilitation, healthcare professionals suggested that they aim to remediate knowledge gaps by learning from colleagues. One nurse suggested that “*it’s only through experience you pick up little things*,” extending this by suggesting that “*you’re learning off other people, who’s to say my way is the right way?*” (Healthcare Professional 3, lines 321–322). This demonstrates the willingness of healthcare professionals to be challenged on aspects of their practice and highlights a worrying dichotomy that healthcare professionals are relying on more experienced healthcare professionals to help remediate perceived knowledge gaps whilst acknowledging that the team does not currently have the depth of understanding of behavior change.

Healthcare professional 3 further stated the utility of her role in supervising nursing students in avoiding “*getting stuck*”: *“if I have a student, I say tell me things because I’ve been doing this (job) now for years”* (Healthcare Professional 3, lines 324–325), further elucidating her willingness to be challenged on aspects of her practice. Similarly, an exercise physiologist reported that she perceived behavior change to be a strength of hers, whilst acknowledging that experiential learning has largely underpinned this understanding: “*Changing a patient’s behavior is probably quite a good strength of mine, that I know how to trigger somebody. That’s just through experience, I’ve got 15 years of doing this*” (Healthcare Professional 5, lines 299–301). Collectively, these claims suggest that healthcare professionals rely upon their experience of helping patients to change behavior rather than drawing on formalized training programs to increase their competence in delivering behavior change and addressing the psychological elements of cardiac and pulmonary rehabilitation within their practice.

### 3.2. Knowledge Translation

The notion that healthcare professionals typically rely on their colleagues to remediate knowledge gaps related to behavior change is problematic. For example, an exercise physiologist reported that “*most of our staff members haven’t got the history in behavior change or psychology*” (Healthcare Professional 5, line 204), and another suggested that “*I’ve got the theory, but the practice, I don’t know. The psychological side for me is neglected a little bit*” (Healthcare Professional 6, lines 273–274). In this instance, the healthcare professional suggests that his theoretical understanding of the psychological elements of rehabilitation stems from his previous academic study. His understanding of behavior change and psychology is, therefore, largely theoretical, with little understanding pertaining to how such concepts could be translated into his practice. This was a commonality between several healthcare professionals, with a largely academic understanding of different theories of behavior change accompanied by a limited understanding of how these models could affect practice. Therefore, an intervention should aim to remediate this knowledge gap by providing a broader and contemporary theoretical basis of the intervention applied to the cardiac and pulmonary rehabilitation service before operationalizing the theory so that healthcare professionals clearly understand how they can translate the theory into their standard clinical practice.

Even when CPD courses were perceived to be relevant, professionals reported a lack of awareness of how to translate these principles into their own practice: “*we know about motivational interviewing, but actually putting it into place, it’s difficult*” (Healthcare Professional 10, lines 333–334). This was also reflected by an exercise physiologist who suggested that motivational interviewing “*was a good course. Very limited that we’ve been able to use anything that was on that course because of the time. Very, very limited*” (Healthcare Professional 6, lines 899–901). In both instances, time pressures within the clinical setting were perceived to limit the extent to which the content of such courses could be translated into healthcare professionals’ everyday practice. Although motivational interviewing was perceived to be a valuable tool for healthcare professionals, it is also demanding, meaning that it was difficult for healthcare professionals to simultaneously deliver an extensive motivational conversation whilst meeting the task requirements that are mandatory within their weekly consultations with patients. This was further elucidated by healthcare professional 6:

*“For me it (ideally) would be to use those skills I want to say now, in the time that we’ve got to be able to use them … the effective things that you can use in ten minutes, that for me would be the gold dust”*

(Healthcare Professional 6, lines 904–908)

Collectively, such quotes demonstrate that CPD courses that equip healthcare professionals with the skills to deliver behavior change and motivational interviewing should be developed in a manner that is mindful of the time constraints associated with clinical settings. For example, a motivational interviewing course should not simply equip healthcare professionals with the skills to ask open questions and make affirmations but should seek to equip professionals with the ability to deliver short behavior change interventions that can be embedded within clinical encounters. This further reinforces the points highlighted by healthcare professional 6, who suggested that he largely understands the “*what?*” of behavior change, indicative of the theoretical underpinnings of behavior change theories. However, the “*how?*” is largely missing within existing CPD opportunities, meaning that the extent to which healthcare professionals have been equipped with the skills and techniques that would allow them to embed behavior change within their practice is limited.

Therefore, interventions need to equip healthcare professionals with a theoretical understanding of behavior change, allowing them to understand why and when they may initiate specific behavior change techniques. Second, interventions should explain the “how?” of behavior change to a larger extent, equipping healthcare professionals with the behavior change they can use to remediate issues with patients’ lifestyle behaviors. When developing these interventions, research teams should be mindful of the stringent time constraints within clinical settings. Practically, intervention developers should ensure that examples used to elucidate specific behavior change techniques should demonstrate how healthcare professionals can implement evidence-based techniques in a contextually relevant manner and in a way that is feasible within the constraints of the clinical setting.

### 3.3. Perceptions of Existing Professional Development Programs

This theme explains what attracts healthcare professionals to CPD courses and their perceptions of the value of such courses. Some healthcare professionals highlighted the limited practical utility of existing CPD courses within cardiac and pulmonary rehabilitation and community rehabilitation settings, with this perceived to be a key factor preventing them from engaging with CPD:

*“What puts me off is I feel like I’m so busy in this role, what puts me off is the fact I don’t think I’m going to learn anything … I’ve got 10,000 million things to do and I’m wasting half a day, or a day probably, sitting in a room and learning not much”*

(Healthcare Professional 5, lines 171–177)

In this instance, healthcare professional 5 highlights the number of competing agendas she has within her role, meaning that any CPD she chooses to engage with needs to benefit one or more of the roles she undertakes for her to perceive it as a productive use of her time. In a similar vein, healthcare professionals perceived a lack of practical utility of existing CPD courses within their own practice in community settings:

*“A lot of our training here, our mandatory training because we’re linked to an acute trust, it’s very acute focused. I don’t know whether there was any more that could be done that’s more suitable for community services, community staff. It’s just like the whole day you’re talking about lines and pumps and things I never use”*

(Healthcare Professional 4, lines 336–339)

Healthcare professional 4 suggests that the content of existing training opportunities offered to them do not reflect the nature of the community-based rehabilitation setting, as they are more suitable for healthcare professionals who work within inpatient settings. As such, when developing interventions and training programs for cardiac and pulmonary rehabilitation, teams should ensure that intervention content is relevant to healthcare contexts and professionals so that the key concepts can be translated into standard practice.

Additionally, the practical utility of existing CPD was also questioned because of its limited ability to affect the problematic patient group who do not attend:

*“To be able to capture the patients that aren’t motivated, they have to come. If they’re not motivated, they’re not coming. We just discharge them and we don’t do anything with them. I think it’s really important and it’s really under-utilized within our service and it’s something that we need to address”*

(Healthcare Professional 5, lines 206–210)

In this instance, healthcare professional 5 highlights a population that would be outside the scope of a face-to-face intervention. These patients typically do not attend cardiac and pulmonary rehabilitation, suggesting the program is “not for them” (Healthcare Professional 6, line 63). Standard practice is to discharge these patients; however, this places them at greater risk of readmission to cardiac and pulmonary rehabilitation, as they are unlikely to have made any lifestyle changes that cardiac and pulmonary rehabilitation would equip them with the skills and level of understanding to achieve. Therefore, it is beneficial to investigate the process a patient progresses through into cardiac and pulmonary rehabilitation and the factors that may lead to them failing to uptake the rehabilitation. From an intervention development perspective, there is a better need to develop interventions that can influence the earlier stages of cardiac and pulmonary rehabilitation such as the inpatient stage, in which healthcare professionals may be better able to influence the likelihood of a patient attending cardiac and pulmonary rehabilitation by exploring reasons for non-attendance or patients’ levels of motivation for engaging in and self-initiating their rehabilitation.

### 3.4. Barriers and Facilitators to Continued Professional Development

Healthcare professionals discussed different factors that acted as a barrier to or facilitator of participating in CPD courses. For example, maintaining a work-life balance was perceived to be a barrier to engaging with CPD:

*“My family life to be honest with you (prevented me from doing CPD), because people have said how about doing your Masters but I found it really hard studying and doing a degree and working full-time and having a young family, it is so hard and draining and I’ve kind of hit a point where I’ve been there and done that, I’ll do what I need to do to keep myself up to date and working, but I’m not going to do any more long-haul stuff because I’ve got two young boys at home and it’s absolutely draining”*

(Healthcare Professional 2, lines 54–59)

Healthcare professional 2 demonstrates the potential of developing a ‘short haul’ behavior change intervention that places less emphasis on the staff to complete work in their own time. In this instance, healthcare professional 2 demonstrates that it is essential that the intervention’s value is clear, in that it should be posed as a method of improving an already outstanding service and aims to complement standard practice without being overly time-demanding on healthcare professionals. These sentiments in relation to time constraints preventing healthcare professionals from engaging with CPD was highlighted in other interviews:

*“The time it takes because everything—you’re expected to do it. Okay you do the courses in the daytime, but all the work is in your own time. That is hard when you’re working full-time to go home and do that”*

(Healthcare Professional 3, lines 298–300)

In contrast to the *“long-haul”* (line 58) courses that healthcare professional 2 discussed, flexibility in courses and professional education made healthcare professionals more likely to engage: “*I’ve got the cardiology app on my phone, it’s really good. Each week I’ll do three topics for 20 min*” (Healthcare Professional 3, lines 265–267). This demonstrates the potential of an intervention that healthcare professionals can dip into in smaller chunks when they perceive the need to, without the need for an extensive formalized education program. Additionally, financial constraints made CPD inaccessible: “*I’ve tried to do leadership loads of times, but I haven’t got the money to fund these courses*” (Healthcare Professional 3, lines 288–290). Financial barriers to CPD were also reflected by another professional who suggested that “*it’s not a cost to us but you’ve got to think of the cost to the service as well*” (Healthcare Professional 6, line 877). These claims reinforce the need to demonstrate CPD value by making it clear why healthcare professionals should commit time and resources.

Collectively, this theme demonstrates that there is a perception that professionals are unable to access CPD courses that they believe would be beneficial due to several factors. Additionally, and most notably, existing CPD courses are not perceived to be tailored to community settings, meaning that healthcare professionals suggest that they have been unable to implement the topics discussed on these courses within their practice. This, therefore, demonstrates the necessity of training programs that are tailored specifically to the community settings, which more readily address problems that community staff face.

## 4. Discussion

The present study investigated healthcare professionals’ experiences of utilizing behavior change interventions in the delivery of cardiac and pulmonary rehabilitation programs. Salient findings from our study found that behavior change in cardiac and pulmonary rehabilitation is an underdeveloped component. Furthermore, healthcare professionals did not perceive themselves to have the competence to deliver effective behavior change interventions as part of their standard practice and did not have access to training programs that could remediate this knowledge gap.

Behavior change is a central component for an effective cardiac and pulmonary rehabilitation service for developing patients’ abilities to self-manage their condition with the aim of preventing readmission to the service [17]. Healthcare professionals in the current study also perceived behavior change to be central to an effective cardiac and pulmonary rehabilitation service. However, healthcare professionals demonstrated a limited understanding of how to effectively integrate behavior change techniques into their practice. Similarly, previous research has found that healthcare professionals are less likely to engage in discussions with patients about health-related behavior change due to feeling unskilled in the area and lacking the confidence to address behavior change issues [18].

In the present study, healthcare professionals perceived training programs and courses that would enable them to improve their understanding and practice of behavior change to be largely inaccessible and lacking practical utility that would facilitate translation. The exception to this was motivational interviewing training, which several healthcare professionals had accessed. The practical application of motivational interviewing upon behavior change and adherence to exercise in the context of cardiac and pulmonary rehabilitation is not well understood. Consequently, there is little current guidance to support the training of healthcare professionals, which is considered an essential component of motivational interviewing for intervention fidelity [19,20]. More generally, it is suggested that there is a lag between behavior change research being conducted and its translation into routine practice [21]. This claim has been elucidated within the broader applied health research field, in which numerous publications have suggested that despite the importance of finding effective ways to encourage healthcare professionals to routinely embed high-quality clinical evidence into their everyday work, this translation has proved a major challenge [21].

Recent research has found that online training has the potential to improve healthcare professionals’ engagement in behavior change conversations with patients [18]. However, as our study has demonstrated, there appears to be no consistent approach for behavior change training and its implementation in practice. NHS provider organizations in England do offer some behavior change training to healthcare professionals in the form of the Making Every Contact Count (MECC) approach [22]. Despite the MECC being a mandated training offer within NHS organizations, healthcare professionals’ levels of engagement with and awareness of MECC are low, and even when healthcare professionals perceive a patient benefit, they do not use MECC behavior change principles in 50% of cases [23]. Additionally, there is a paucity of evidence to support MECC’s effectiveness, both in terms of professional behavior change training [24] and patients making changes to their health-related behaviors [18]. In addition, those responsible for designing and delivering health behavior education for healthcare professionals are unclear about what to include, and even because those who teach trainees about behavior change struggle with delivering behavior change as part of their own practice [8].

It is important to recognize that the findings from this study are contained to a relatively small number of participants and are specific to a particular context. However, by positioning the present study’s findings within a health system setting, it was evident that a greater understanding of the training needs for both healthcare professionals and those commissioning behavior change training is required. Greater attention toward behavior change training can help upskill healthcare professionals’ clinical practice and competence for delivering behavior change interventions. However, at present, there is a paucity of research investigating existing behavior change practices within healthcare services. Future research in this area may wish to consider adopting a particular methodological-oriented qualitative form of inquiry (e.g., phenomenology), given that the current study utilized a thematic approach, which is known to be a broad method for conducting qualitative analysis [25].

## 5. Conclusions

Our findings provide a contextualized understanding of behavior change for healthcare professionals working in a cardiac and rehabilitation service in the UK. Currently, training programs enhancing healthcare professionals’ levels of understanding and competence in delivering behavior change were perceived to be inaccessible. To exacerbate this, current behavioral change training was not perceived to equip healthcare professionals with an extensive appreciation of how to translate behavior change into their routine practice within cardiac and pulmonary rehabilitation. The development and evaluation of behavior change training programs have the potential to benefit healthcare professionals. The current study provides a useful starting point for understanding healthcare professionals’ views of behavior change in cardiac and pulmonary rehabilitation.

## Data Availability

Data are available upon request due to privacy/ethical restrictions.
